# Morphological, physiological, and transcriptomic analysis of *Taxodium mucronatum* under different salinity stresses

**DOI:** 10.3389/fpls.2026.1686191

**Published:** 2026-02-04

**Authors:** Yunpeng Gao, Hongling Wang, Shizheng Shi, Ruifang Huang, Liwen Liang, Jue Zhang, Kaipeng Jiang, Tao Huang, Shuxian Li, Cong Lei, Yawen Dai, Dezong Sui

**Affiliations:** 1 Institute of Genetic Breeding, Jiangsu Academy of Forestry, Nanjing, Jiangsu, China; 2 College of Forestry and Grass, Nanjing Forestry University, Nanjing, Jiangsu, China; 3 Southern Modern Forestry Collaborative Innovation Center, Nanjing Forestry University, Nanjing, Jiangsu, China

**Keywords:** antioxidant enzyme, osmoprotectants, salt stress, *Taxodium mucronatum*, WGCNA

## Abstract

**Introduction:**

Soil salinity is a pressing global issue that undermines agricultural productivity, driving the search for salt-tolerant species and their adaptive strategies. *Taxodium mucronatum*, a tenacious afforestation tree species, is known for its notable resistance to abiotic stresses. However, its molecular response to salt stress is still unknown.

**Methods:**

In this study, we explored the physiological and transcriptomic adaptations of *T. mucronatum* seedlings when exposed to different NaCl concentrations (0 ‰, CK; 3 ‰, LS; 5 ‰, MS; 7 ‰, HS).

**Results and discussion:**

Through morphological and biochemical analyses, we identified a salinity threshold of 5 ‰. Beyond this threshold, severe leaf senescence and plant death were observed. In physiological profiling, the malondialdehyde (MDA) and relative conductivity (REL) showed dose-dependent increases. Meanwhile, osmoprotectants like proline (PRO), soluble sugar (SS), and soluble protein (SP), as well as antioxidant enzyme activities including peroxidase (POD), catalase (CAT), and superoxide dismutase (SOD), were elevated. This indicates dynamic responses to osmotic and oxidative stress. Transcriptome sequencing revealed 3,858 differentially expressed genes (DEGs). GO and KEGG analyses showed that the commonly up-regulated genes were enriched in ‘oxidoreductase activity’ (GO:0016491) and ‘phenylpropanoid biosynthesis’ (ko00940), whereas down-regulated genes were enriched in ‘cell-wall organization’ (GO:0071554). Among the 421 differentially expressed transcription factors, ERF, WRKY and NAC families constituted 62% of the total, indicating their central role in the salt response. With Weighted Gene Co-expression Network Analysis (WGCNA), we first linked gene modules to physiological traits and found that the MEbrown (r = 0.67–0.99) positively and MEblue (r = –0.69 to –0.98) negatively drives osmoprotectant/antioxidant activation. From these modules, 12 hub genes —especially *TCTP*, *ECI3*, *PGL3*, *OsI_15387*, *APF2*, *CYP73A4*— were identified that coordinate stress adaptation via cell wall remodeling, energy metabolism, and redox homeostasis. This study offers the first in-depth analysis of salt tolerance mechanisms in *T. mucronatum*, revealing genotype-specific strategies to cope with ionic and osmotic stress. The findings enhance our molecular understanding of stress resilience in woody perennials and highlight the potential for ecological restoration of *T. mucronatum* in saline-alkali ecosystems.

## 1 Introduction

Rising soil salinity is emerging as a critical environmental challenge, imposing considerable constraints on agricultural productivity. This issue is especially evident in arid and semi-arid regions, where the accumulation of sodium chloride (NaCl) in the soil is a key concern (El Sabagh et al., 2020). Globally, excessive salinity affects over 900 million hectares of land, including regions in Asia, Africa, Australia, and the Middle East (van Zelm et al., 2020). The problem is exacerbated by global warming and human activities, with annual losses from salinization amounting to as much as 27.3 billion dollars (Qadir et al., 2014). In irrigated regions, the situation is even more severe, with approximately 20% of the area affected by salinity, thereby posing a direct threat to global food security (Balasubramaniam et al., 2023).

The presence of salts in the soil primarily affects plants through osmotic stress. Osmotic stress arises because elevated salt concentrations lower the soil water potential, thereby curtailing the plant’s ability to absorb water (Bhattacharya, 2021). Salt stress has been demonstrated to induce oxidative stress, which is characterized by the overproduction of reactive oxygen species (ROS). Consequently, damage to cellular components, including lipids, proteins, and DNA, has been observed (Ahmad et al., 2019), resulting in growth inhibition. During this process, plasma-membrane integrity is markedly compromised, as indicated by increased electrolyte leakage and relative conductivity (REL), together with elevated malondialdehyde (MDA) levels that signify intensified membrane lipid peroxidation (Zhang et al., 2020). To cope with salt stress, it has been observed that plants accumulate osmoprotectants such as proline (PRO), soluble sugars (SS), and soluble proteins (SP) to restore osmotic balance and maintain ion homeostasis. Furthermore, in order to counteract various biotic and abiotic stresses, plants have evolved a complex antioxidant system over the course of their evolution. The system is responsible for the regulation of ROS homeostasis, which comprises enzymes such as superoxide dismutase (SOD), catalase (CAT), and peroxidase (POD) (Liang et al., 2018).

The advent of sequencing technologies has engendered a paradigm shift in the utilization of transcriptome sequencing data for the exploration of metabolic pathways associated with plant responses to salt stress, thereby facilitating the identification of candidate salt-tolerance genes (Zhu et al., 2025). Transcriptome sequencing has been used to investigate the salt-tolerance mechanisms of crops such as rice and soybean (Fang et al., 2023). However, the majority of current research is focused on temporal dimension mechanisms, with fewer studies on responses to different salt concentrations. It has been established that under varying degrees of salt stress, a greater number of differentially expressed genes (DEGs) are enriched in categories pertaining to metabolic processes, catalytic activity, and membrane components (Deng et al., 2025). Weighted gene co-expression network analysis (WGCNA) is a widely utilized approach for identifying pivotal regulatory genes and predicting the functions of uncharacterized genes (Wan et al., 2018). This methodology has been demonstrated to substantially enhance the understanding of salt stress response in plants (Ju et al., 2023). Moreover, the levels of transcription factors (TFs) such as ERF and WRKY are subject to change in response to varying salt concentrations, thereby facilitating dynamic transcriptional regulation (Zhu et al., 2025).

*Taxodium mucronatum* is a valuable afforestation tree species, characterized by its rapid growth, significant adaptability, and notable tolerance to abiotic stresses (Arroyo et al., 2011). The cultivation of crops on saline soils has been identified as a significant strategy for ameliorating saline conditions (Öztürk et al., 2025). In this study, we used different concentrations of NaCl solutions to simulate salt stress and explored the salt tolerance threshold of *T. mucronatum*. Osmoprotectants and antioxidant enzyme activities were measured to elucidate specific responses at various salt concentrations. By comparing transcriptome analyses, we examined differences in gene expression profiles between different salt treatment concentrations. To integrate the two data layers, we performed WGCNA to identify gene modules significantly associated with the measured physiological–biochemical traits and thereby pinpoint key functional modules and hub genes underlying salt-stress adaptation. The objective of this study is to reveal the mechanisms of osmoregulation and antioxidant stress response through the correlation analysis of physiological and biochemical indicators and transcriptome. This study will facilitate a more profound comprehension of the molecular mechanisms of *T. mucronatum* in response to salt stress, thereby enabling further exploration of its potential application value in saline-alkali lands.

## 2 Materials and methods

### 2.1 Plant material and samples collection

The experiment was conducted in a glasshouse at the Lishui Experimental Base of Nanjing Forestry University (31.52° N, 119.18° E) using 2-year-old potted *T. mucronatum* seedlings (provenance: seed orchard of Jiangsu Academy of Forestry, 31°52′ N, 118°48′ E, Jiangsu, China). The seedlings were cultivated in a 1:1 (v/v) mixture of nutrient soil (patent ZL201310709279.6) and garden soil (0–15 cm; pH 6.08, total N 7.34 g·kg^-1^, total P 0.28 g·kg^-1^, total K 74.01 g·kg^-1^). Glasshouse conditions were maintained at 25 ± 2 °C day/22 ± 2 °C night, 65–75% RH, and 1,000 ± 100 µmol m^-2^ s^-1^ PAR (14 h photoperiod) with automatic daily monitoring. In July 2024, healthy and uniformly vigorous seedlings were subjected to salt stress treatments with three salinity levels (LS (3 ‰), MS (5 ‰), and HS (7 ‰)), which correspond to soil electrical conductivities of 1200±50, 1900±50, and 2500±50 μS/cm, respectively. The control group was treated with pure water (0 ‰). To prevent salt leaching, trays were placed under each pot. Additionally, soil electrical conductivity (EC)was monitored and adjusted every three days via 1:5 soil to water ratios to keep each treatment within its designated EC range (He et al., 2012). The experiment was performed on three replicates with 20 seedlings per replicate to ensure statistical significance.

Following a 15-day period, morphological assessments were conducted, and samples from the CK, LS, and MS treatments were collected (HS treatment resulted in plant death). For each biological replicate, the 2nd and 3rd fully-expanded needle fascicles (from the shoot apex) were collected from ten randomly selected seedlings; the middle segment of each needle was taken, pooled per replicate, immediately flash-frozen in liquid nitrogen, and stored at −80 °C for further analyses.

### 2.2 Morphological observation and determination of stress level indicators

Morphological observations were conducted using a high-resolution camera (Canon EOS 80D, Japan) to document the above-ground condition of each treatment. Leaf samples (0.20 g, fresh weight) from each treatment were weighed to determine RELand MDA content, with three independent biological replicates analyzed per treatment. The REL was measured using a precise conductivity meter to assess the integrity of the cell membranes. Additionally, the thiobarbituric acid (TBA) method was employed to measure the MDA content, which indicates lipid peroxidation and oxidative damage (Li and Zhang, 2025).

### 2.3 Determination of osmoprotectant contents and antioxidant enzyme activities

Leaf samples (0.20 g, fresh weight) from each treatment were weighed for the determination of osmoprotectant contents and antioxidant enzyme activities, with three independent biological replicates analyzed per treatment. The osmoprotectant composition, encompassing PRO, SS, and SP, was analyzed. The SS content was determined in accordance with the methods outlined by Zhu et al. (2024) employing anthrone colorimetry. The SP content was assessed using the Coomassie Brilliant Blue assay (Zhu et al., 2025), and the PRO content was measured following the method established by Bates et al. (1973), with some adjustments by Abdelrady et al. (2024).

The activities of antioxidant enzymes, including POD, CAT, and SOD, were evaluated in order to assess the antioxidant defense mechanisms. The prepared sample was then utilized for the analysis of SOD activity, employing the nitroblue tetrazolium (NBT) method. The resultant data were then read at 560 nm (Giannopolitis and Ries, 1977). The activity of POD was determined spectrophotometrically in a reaction mixture containing guaiacol, H_2_O_2_, K-P buffer with EDTA (pH 7.0), by measuring the absorbance at 470 nm using a spectrophotometer. CAT activity was assayed by monitoring the absorbance at 240 nm in the same buffer (Ali et al., 2021).

### 2.4 RNA extraction, library construction, and RNA−seq

For transcriptome sequencing (RNA-seq), three biological replicates were collected from each of the CK, LS, and MS treatments. The generation of RNA-seq libraries involved a total of nine samples (three treatments × three biological replicates). Total RNA was isolated with the RNAprep Pure Plant Kit (DP441, Tiangen Biotech, Beijing, China) following the manufacturer’s protocol v4.3. Strand-specific libraries were constructed using the NEBNext^®^ Ultra™ II RNA Library Prep Kit for Illumina^®^ (E7775L, New England Biolabs, USA) and indexed with NEBNext Multiplex Oligos (E7600S). Paired-end 150-bp sequencing was performed on an Illumina NovaSeq 6000 platform (Illumina Inc., USA) at Gene Denovo Biotechnology Co., Guangzhou, China. All raw data have been deposited in the National Genomics Data Center (NGDC), China National Center for Bioinformation, under project number PRJCA052949.

### 2.5 *De novo* assembly and functional annotation

The Fastp software was utilized to regulate the quality of the offline raw reads and eliminate those of substandard quality, i.e., those containing more than 10% unknown nucleotides (N) and those containing more than 50% low-quality bases (Q value ≤ 20). The objective of this process was undertaken to obtain a refined dataset. The unigenes were obtained by means of sequence assembly using Trinity. In order to annotate the unigenes, the BLASTx program (http://www.ncbi.nlm.nih.gov/BLAST/) was utilized with an E-value threshold of e^-5^ in order to facilitate comparison against a number of databases, including Nr (NCBI non-redundant protein sequences), Swiss-Prot, KEGG (Kyoto Encyclopedia of Genes and Genomes), GO (Gene Ontology), COG/KOG (Clusters of Orthologous Groups) and Pfam (Protein family). Protein functional annotations could then be obtained according to the best alignment results.

### 2.6 TPM calculation and PCA analysis

Utilizing the Trinity assembled transcriptome as a reference sequence, the clean reads from each sample were aligned using the RSEM software to calculate TPM (Transcripts Per Kilobase of exon model per Million mapped reads). The assessment of inter-sample reproducibility was conducted on the basis of the expression results of each sample. To this end, Principal Component Analysis (PCA) was employed, utilizing the R package models (http://www.r-project.org/).

### 2.7 GO and KEGG analyses of DEGs

The differential expression analysis of RNA was conducted using DESeq2 software, comparing two distinct groups. The genes exhibiting a false discovery rate (FDR) below 0.05 and an absolute fold change of at least 2 were designated as differentially expressed genes (DEGs). The GO and KEGG databases were utilized to perform enrichment analyses of transcripts and DEGs per sample. The hypergeometric test was utilized to identify GO terms and significantly enriched pathways among the DEGs when compared to the genomic background. Subsequently, the resultant *P*-values were then adjusted, with an FDR ≤ 0.05 considered significant.

### 2.8 Identification of potential TFs

Protein coding sequences of unigenes were aligned by BLASTp to PlantTFdb (http://planttfdb.cbi.pku.edu.cn/) to predict TF families. The e-value was set as the default value, and the number of genes annotated to TF families was counted.

### 2.9 Weighted gene co-expression network analysis

WGCNA was conducted on the basis of expression correlation patterns. The genes were analyzed using the log_2_-transformed TPM values plus one as input, and the soft thresholding power was determined by the scale-free network criterion. The lowest power value at which the scale independence reached a plateau (0.85) was selected for downstream analysis, and the changes in gene connectivity at various power values were also examined. The genes were then organized into modules using a dynamic tree-cutting approach. A gene clustering dendrogram was constructed on the basis of gene expression correlations, and gene modules were defined according to the clustering dendrogram. Modules exhibiting analogous expression profiles were subsequently amalgamated, with the premise that modules with a minimum of 50 genes and a merging threshold of 0.8 would demonstrate sufficient similarity. The significance of modules was determined through module eigengene analysis, and relevant modules were selected for further investigation. Finally, the top 20 connectivity relationships of each significant module were selected for display, and the hub genes of each module were determined by weight values.

### 2.10 qRT-PCR validation

For qRT-PCR, 1 μg of total RNA was reverse-transcribed using the PrimerScript™ RT reagent Kit with gDNA Eraser (RR047, Takara, Japan). qRT-PCR was then carried out on a LightCycler 480 Real-Time PCR System (Roche, Basel, Switzerland) in a 10 μl volume containing 5.0 μl of SYBR Premix ExTaq™ (RR820A), 0.5 μl of each primer (10 μM), 2 μl of cDNA and 2 μl of ddH_2_O. The PCR conditions were described in detail in Chen et al. (2024). *GAPDH* was used as an internal control. Primers were designed using Primer 5.0 software (**Supplementary Table 1**). Each reaction was performed with three biological replicates, with each sample analyzed in triplicate. Gene expression levels were calculated using the 2^−ΔΔCT^ method.

### 2.11 Statistical analysis

Data were organized in Microsoft Excel 2013. Analysis of variance (ANOVA) and *t*-test were conducted in SPSS 19.0. Graphs were generated using Origin 2018 and final images were prepared in Adobe Illustrator 2022.

## 3 Results

### 3.1 Impact of salt stress on growth and membrane stability

**Figure 1:**
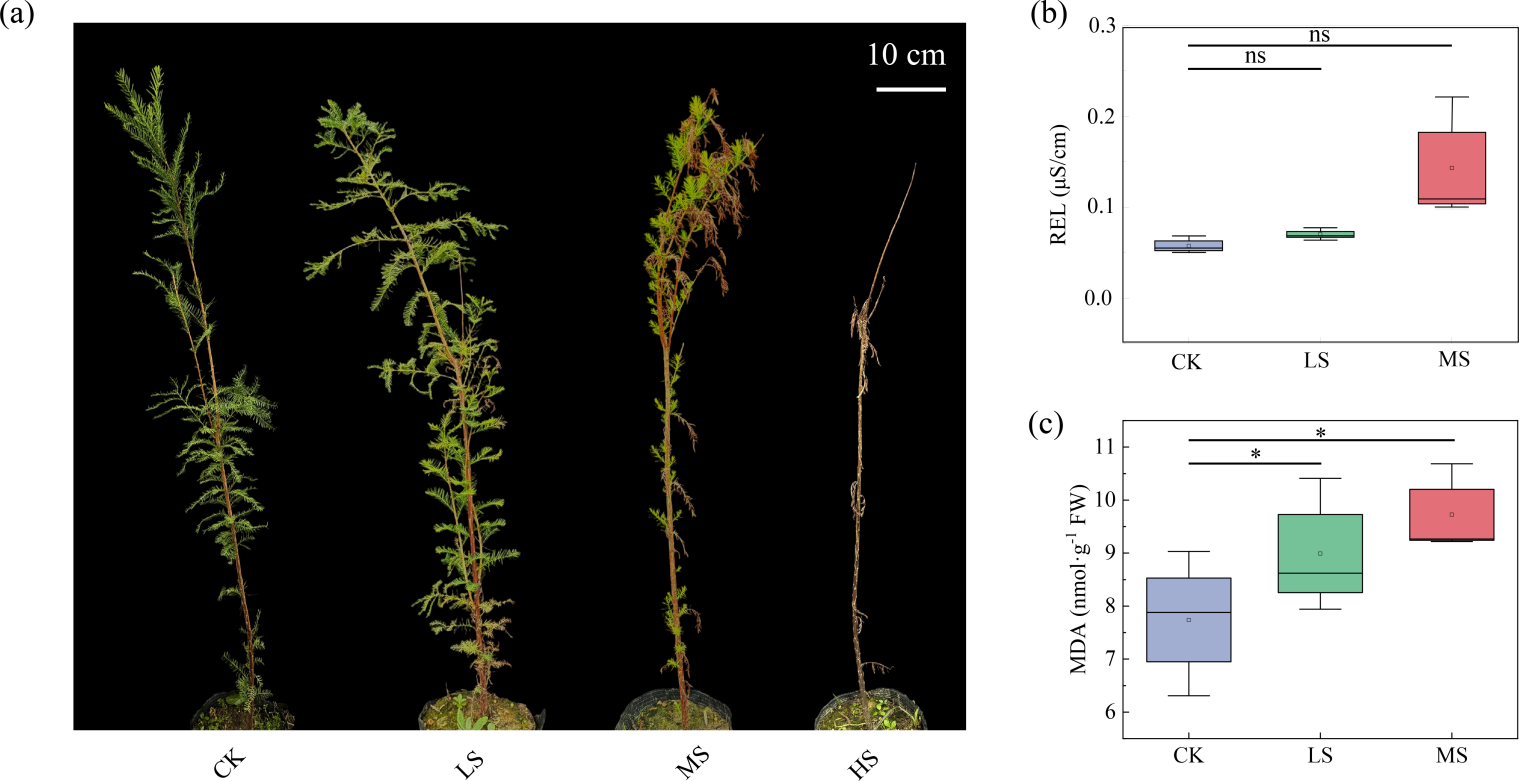
Responses of *T. mucronatum* to different salt stress treatments, including **(A)** phenotypic characterization, **(B)** relative conductivity, and **(C)** MDA content. Different letters indicate significant differences by *t*-test. The symbols ns indicate no significant difference, *P* > 0.05; * indicate significant differences at *P* < 0.05 and *P* < 0.01 levels, respectively. Error bars represent the standard error of the mean. CK indicates the control group; LS, MS, and HS indicate 3 ‰, 5 ‰, and 7 ‰ salt stress treatment groups, respectively.

To visually and quantitatively evaluate the salinity tolerance threshold of *T. mucronatum*, we first documented above-ground morphological changes and then assessed cell membrane integrity under increasing NaCl concentrations. The growth vigor of *T. mucronatum* exhibited a progressive decline with increasing NaCl concentrations (**Figure 1A**). Specifically, the LS treatment induced minimal leaf senescence, whereas the MS treatment caused pronounced leaf senescence accompanied by shoot-tip dieback. Complete plant mortality was observed under HS treatment.

The NaCl-induced morphological deterioration was associated with escalating cellular damage, as evidenced by the dose-dependent rises in REL and MDA content (**Figures 1B, C**). While the REL did not differ significantly between the CK and either LS or MS treatments, the MDA content increased significantly by 1.48-fold (LS) and 1.6-fold (MS) compared to the CK, respectively.

### 3.2 Effects of salt stress on osmoprotectants and antioxidant enzymes

**Figure 2:**
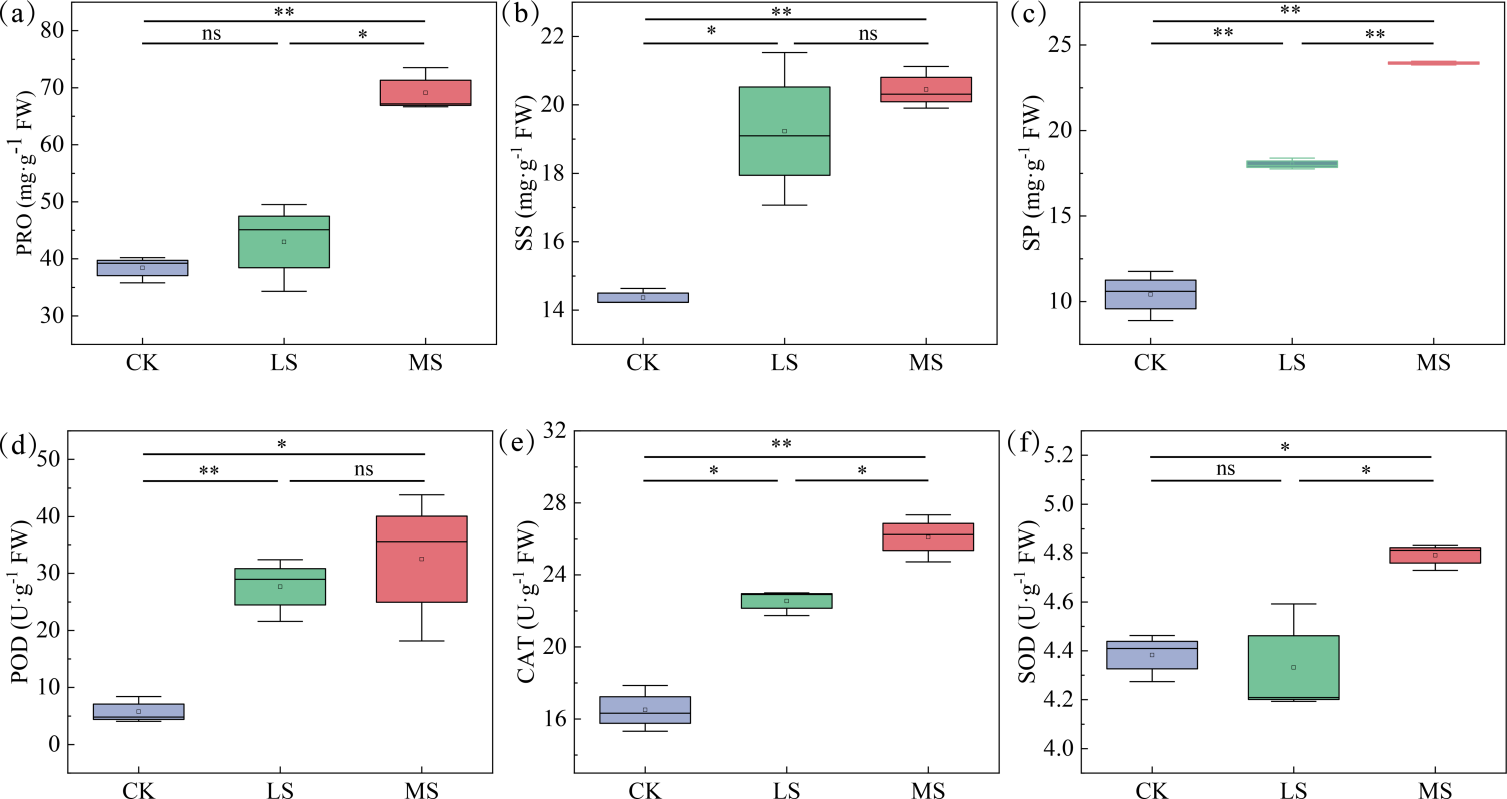
Effects of different salt stress treatments on osmoprotectants and antioxidant enzymes in *T. mucronatum*. **(A)** PRO content; **(B)** SS content; **(C)** SP content; **(D)** POD activity; **(E)** CAT activity; **(F)** SOD activity. Different letters indicate significant differences by *t*-test. The symbols ns indicate no significant difference, *P* > 0.05; * and ** indicate significant differences at *P* < 0.05 and *P* < 0.01 levels, respectively. Error bars represent the standard error of the mean. CK indicates the control group (0 ‰ NaCl); LS, MS, and HS indicate 3 ‰, 5 ‰, and 7 ‰ salt stress treatment groups, respectively.

To uncover the physiological mechanisms underlying the observed phenotype, we quantified key osmoprotectants and antioxidant enzyme activities that maintain osmotic and redox homeostasis. Physiological profiling revealed a striking salt concentration-dependent modulation of both osmoprotectants and antioxidant enzyme activities (**Figure 2**). The contents of osmoprotectants, including Pro, SS, and SP, increased significantly with NaCl concentration (**Figures 2A–C**). Under MS treatment, Pro content surged to 69.12 mg·g^-1^ (1.79-fold increase over CK), while SS and SP reached 20.45 mg·g^-1^ and 23.94 mg·g^-1^, representing increases of 1.42-fold and 2.30-fold, respectively. All osmoprotectants reached their peak levels under this treatment. Importantly, SS and SP contents also displayed significant elevation under LS stress compared to CK. Specifically, SS content increased from 14.36 mg·g^-1^ in CK to 19.23 mg·g^-1^ under LS, and SP content rose from 10.42 mg·g^-1^ to 18.03 mg·g^-1^.

Antioxidant enzyme activities also exhibited dose-dependent increases (**Figures 2D–F**). Both POD and CAT activities were significantly induced by salt stress. Under MS treatment, POD activity peaked at 32.50 U·g^-1^ (5.65-fold increase over CK), and CAT activity reached 26.11 U·g^-1^ (1.58-fold increase). In contrast, SOD activity responded differently. It showed no significant change under LS stress, but increased significantly to 4.79 U·g^-1^ (1.09-fold increase) only under the higher MS stress level.

### 3.3 Transcriptome profiling and functional enrichment analysis

To dissect the molecular basis of salt tolerance in a woody conifer, we performed RNA-seq on the same leaf samples used for physiological assays. A total of 364,277,486 raw reads were generated, with 362,811,570 high-quality clean reads retained after rigorous quality filtering. The sequencing data exhibited exceptional quality, with Q20 values ranging from 98.78% to 98.96% and Q30 values from 96.08% to 96.28%, alongside GC content maintained between 43.94% and 44.03% (**Supplementary Table 2**). The metrics thus provide confirmation of the robustness and reliability of the dataset for downstream analysis.

The *de novo* assembly of the transcriptome under salt stress resulted in 29,579 unigenes. A total of 24,837 unigenes were successfully annotated across multiple databases: The number of sequences in GO was 17,479, in KEGG 9,125, in NR 24,220, in SwissProt 16,204, in PFAM 17,603, and in KOG 12,200. It is noteworthy that a total of 6,075 genes were annotated across all six databases (**Supplementary Figure 1**). These annotations provide a comprehensive overview of the functional characteristics of the identified genes.

**Figure 3:**
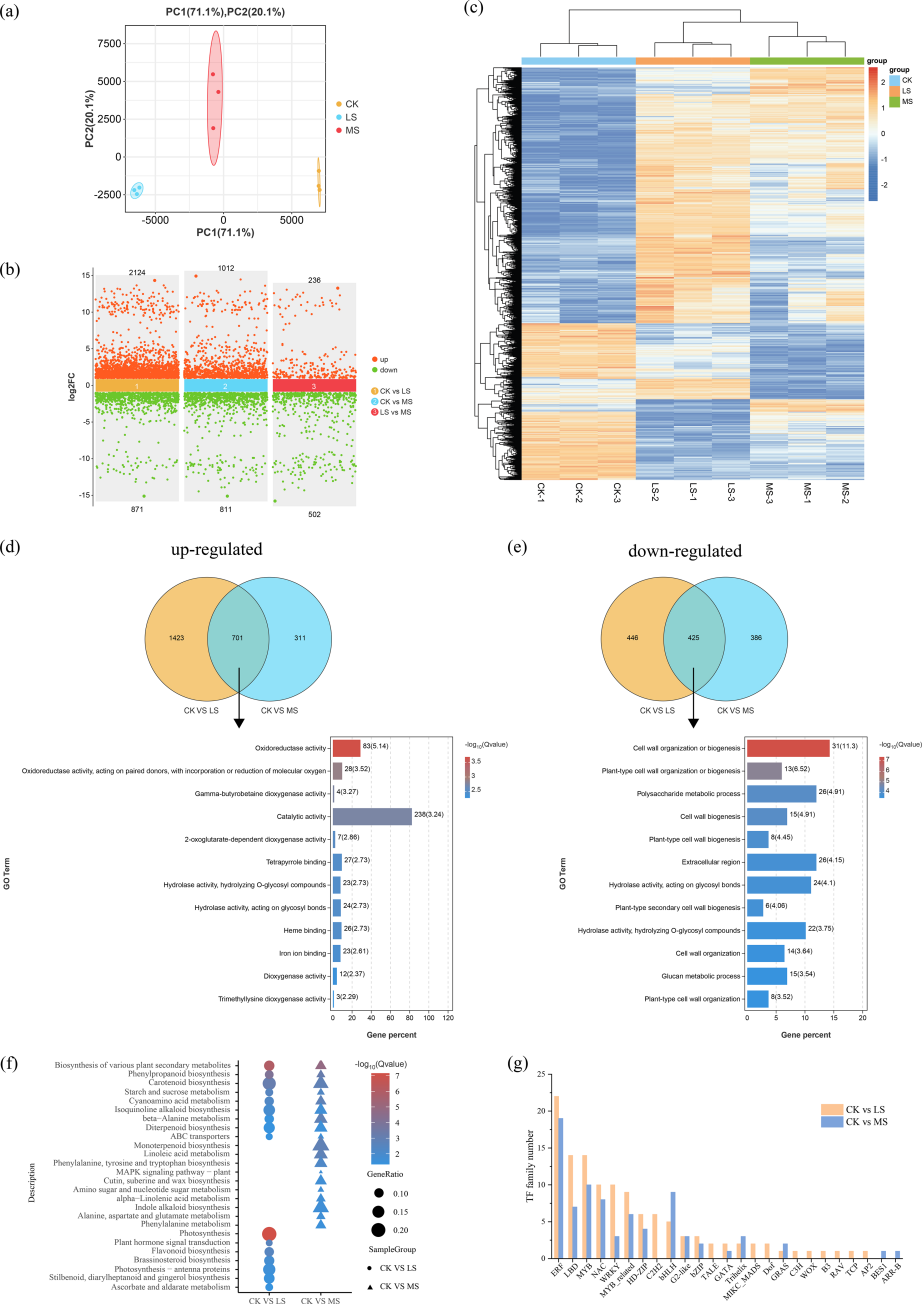
Transcriptomic quality and analysis of differentially expressed genes. **(A)** Principal component analysis. **(B)** Scatter plot of up-regulated and down-regulated DEGs. **(C)** Heatmap analysis of DEGs. **(D)** Venn diagram for the overlap between DEGs in CK and LS treatments, and GO enrichment analysis of the overlapping genes. **(E)** Venn diagram for the overlap between DEGs in CK and MS treatments, and GO enrichment analysis of the overlapping genes. **(F)** KEGG enrichment of DEGs under different salt stress treatments. **(G)** Numbers of DEGs annotated to different transcription factor families under different salt stress treatments. CK indicates the control group (0 ‰ NaCl); LS, MS, and HS indicate 3 ‰, 5 ‰, and 7 ‰ salt stress treatment groups, respectively.

To assess the overall distribution and clustering patterns among the samples, principal component analysis (PCA) was performed on the transcriptomic data. Principal Component 1 (PC1) and Principal Component 2 (PC2) collectively accounted for 91.2% of the total variance, with PC1 capturing 71.1% and PC2 capturing 20.1% of the variation (**Figure 3A**). Notably, the three biological replicates for each treatment exhibited tight clustering, reflecting minimal variation within each group. This consistent clustering pattern underscores the high reproducibility and reliability of the sequencing data.

To quantify the global transcriptional response of *T. mucronatum* to progressive salt stress, we identified differentially expressed genes (DEGs) between control and each treatment. A total of 3,858 DEGs were identified. Pairwise comparisons between CK vs LS, CK vs MS, and LS vs MS revealed 2,995 DEGs (2,124 up-regulated and 871 down-regulated), 1,823 DEGs (1,012 up-regulated and 811 down-regulated), and 738 DEGs (236 up-regulated and 502 down-regulated), respectively (**Figure 3B**). The heat map of all DEGs highlighted that salt stress induced significant transcriptomic changes in *T. mucronatum* (**Figure 3C**).

To uncover the major biological processes driving the early and mid-phase salt response, we performed Gene Ontology (GO) enrichment on the commonly up- and down-regulated DEGs. We compared the upregulated genes between CK vs. LS and CK vs. MS, identifying a total of 701 commonly upregulated differentially expressed genes (DEGs). Upon performing GO enrichment analysis on these shared upregulated DEGs, we found that they were predominantly associated with Oxidation-Reduction Reaction. For instance, the terms Oxidoreductase activity (GO:0016491) and Oxidoreductase activity, acting on paired donors, with incorporation or reduction of molecular oxygen (GO:0016705) were significantly enriched (**Figure 3D**).

Similarly, we identified 425 commonly downregulated DEGs. GO enrichment analysis revealed that these downregulated DEGs were highly enriched in terms related to cell wall construction or modification. Notably, terms such as Cell wall organization or biogenesis (GO:0071554) and Plant-type cell wall organization or biogenesis (GO:0071669) were prominently represented (**Figure 3E**).

To map the DEGs onto defined metabolic pathways and pinpoint primary defense routes, we performed KEGG analysis on the DEGs identified in the comparisons of CK vs. LS and CK vs. MS, revealing common enrichment in pathways related to biosynthesis and metabolism (**Figure 3F**). For example, pathways such as Biosynthesis of various plant secondary metabolites (Ko00999), Phenylpropanoid biosynthesis (Ko00940), and Carotenoid biosynthesis (Ko00906) were found to be enriched. These findings underscore the pivotal functions of these pathways in the response of *T. mucronatum* to salt stress.

Notably, differences in enriched pathways were also observed under different concentration treatments. In the CK vs. LS comparison, pathways such as Photosynthesis (Ko00195) and Flavonoid biosynthesis (Ko00941) were enriched. However, in the CK vs. MS comparison, pathways like Monoterpenoid biosynthesis (Ko00902) and Linoleic acid metabolism (Ko00591) were found to be enriched. The results obtained demonstrate the distinct responses of *T. mucronatum* to varying levels of salt stress.

### 3.4 Identification of potential TFs

To reveal the upstream regulators potentially controlling these defense pathways, we surveyed the differentially expressed transcription factors (TFs). Potential salt stress-regulatory TFs were identified by comparing CK vs. LS and CK vs. MS (**Figure 3G**). The majority of these TFs belonged to the ethylene-responsive factor (ERF), lateral organ boundaries domain (LBD), myeloblastosis (MYB), no apical meristem/ATAF1/2/cup-shaped cotyledon (NAC), and WRKY families. However, the number of TFs from specific TF families varied between tissues. In general, the number of TFs in CK vs. LS was higher than that in CK vs. MS. Notably, TFs from the basic helix–loop–helix (bHLH), trihelix, and gibberellic acid insensitive (GAI), repressor of ga1-3 (RGA) and scarecrow (SCR) (GRAS) families were more abundant in CK vs. MS, while other TF families were more enriched in CK vs. LS. In this study, certain TFs were observed to exhibit elevated expression levels under salt stress conditions (**Figure 3G**).

### 3.5 Construction of gene co-expression modules and identification of hub genes

**Figure 4:**
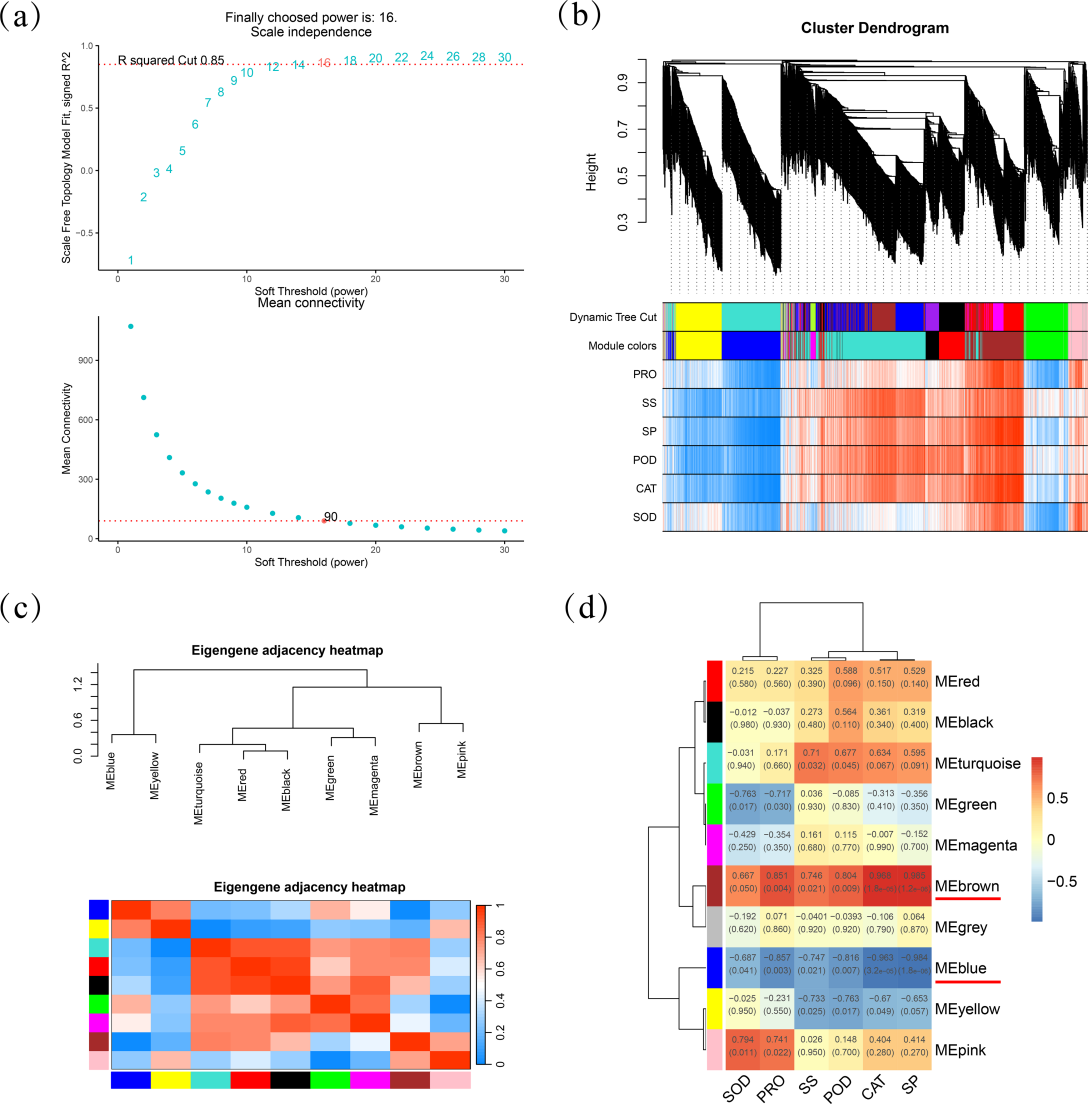
Weighted gene co-expression network analysis (WGCNA) construction and core module identification. **(A)** Power value and network topological fit index relationship curve. **(B)** Module-level clustering. **(C)** Eigengene adjacency heatmap. **(D)** Module-trait correlation heat map.

To move from single-gene lists to systems-level insight, we conducted Weighted Gene Co-expression Network Analysis (WGCNA) by integrating gene expression data with six distinct phenotypic values. When setting the correlation threshold at 0.85, the power value was determined to be 16, with a connectivity of 90 at this point (**Figure 4A**). After merging modules with similar expression patterns, we obtained nine co-expression modules (**Figures 4B, C**). By calculating the correlations between the constructed co-expression modules and each trait, a heatmap was generated to depict the relationships between modules and phenotypic characteristics (**Figure 4D**). Further analysis revealed that the MEbrown exhibited significant positive correlations with all six physiological traits (r = 0.67–0.99), particularly with SP (r = 0.99), CAT (r = 0.97) and Pro (r = 0.85), indicating comprehensive activation of osmoprotective and antioxidant pathways. In contrast, MEblue showed significant negative correlations with these traits (r = –0.69 to –0.98, p < 0.05), suggesting it represents a suppressed state of stress-defense mechanisms.

**Figure 5:**
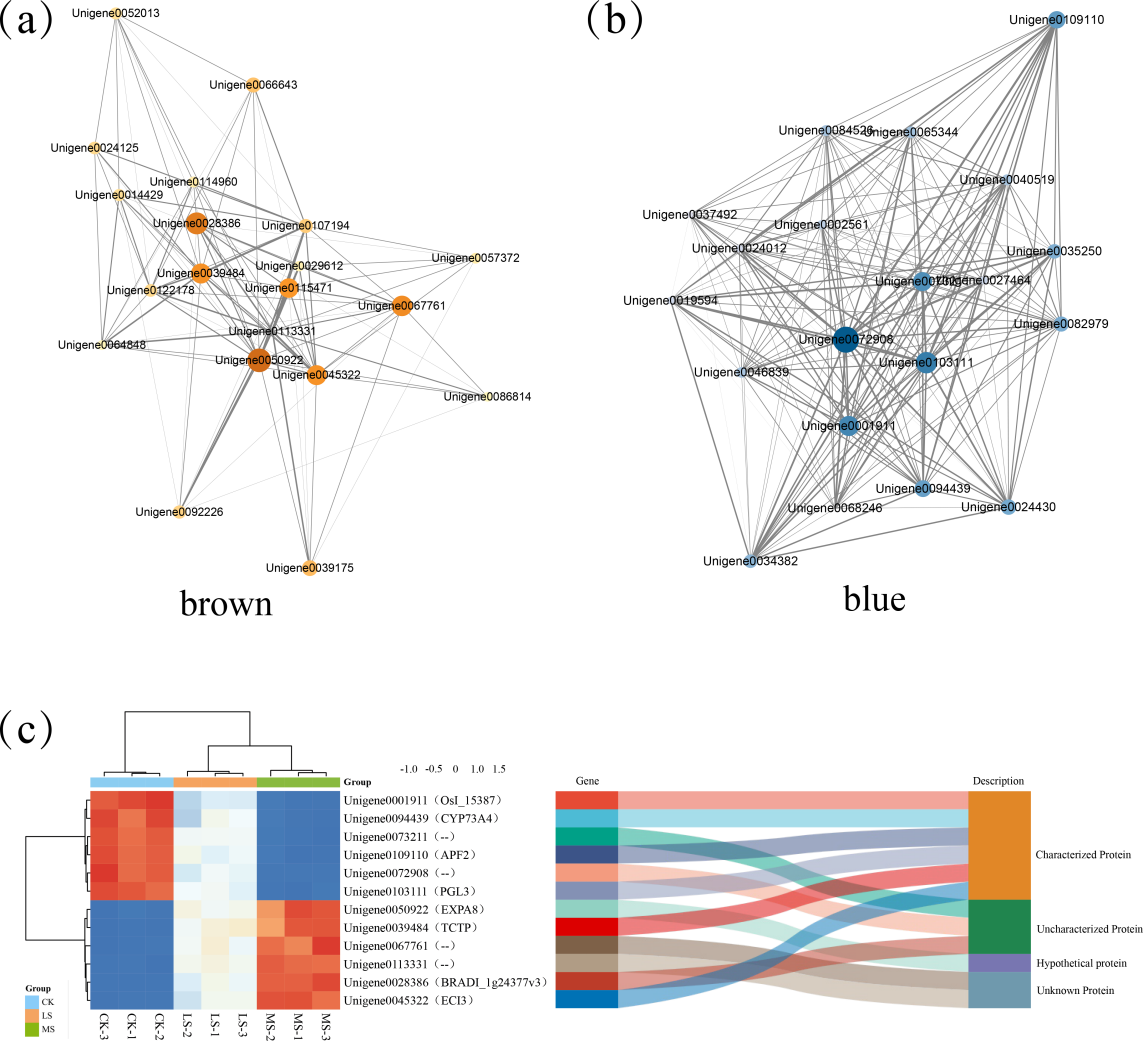
Construction of co-expression network for hub modules and identification of hub genes. **(A)** the ME brown module; **(B)** the ME blue module. The color and size of the bubbles represent the q-values and number of genes, respectively. Thicker lines represent stronger associations. **(C)** Heatmap and classification of hub genes.

Based on the above two hub modules, co-expression networks were constructed to further explore the hub genes, that play a central regulatory role (**Figures 5A, B**). Finally, a total of 12 hub genes were identified from the brown and blue modules. Among them, two genes encode unknown proteins, one encodes a hypothetical protein, and three encode uncharacterized proteins (**Figure 5C**). The remaining six encode characterized proteins, including *TRANSLATIONALLY CONTROLLED TUMOR PROTEIN* (*TCTP*), *ENOYL-COA ISOMERASE3* (*ECI3*), *PHOSPHOGLUCOMUTASE-LIKE3* (*PGL3*), *AN UNCHARACTERIZED ORYZA SATIVA INDICA GROUP PROTEIN* (*OsI_15387*), *AP2/B3-LIKE TRANSCRIPTIONAL FACTOR FAMILY PROTEIN2* (*APF2*), and *CYTOCHROME P45073A4* (*CYP73A4*), which are considered to play a crucial role in *T. mucronatum* responding to salt stress.

### 3.6 Verification of gene expression through qRT−PCR

**Figure 6:**
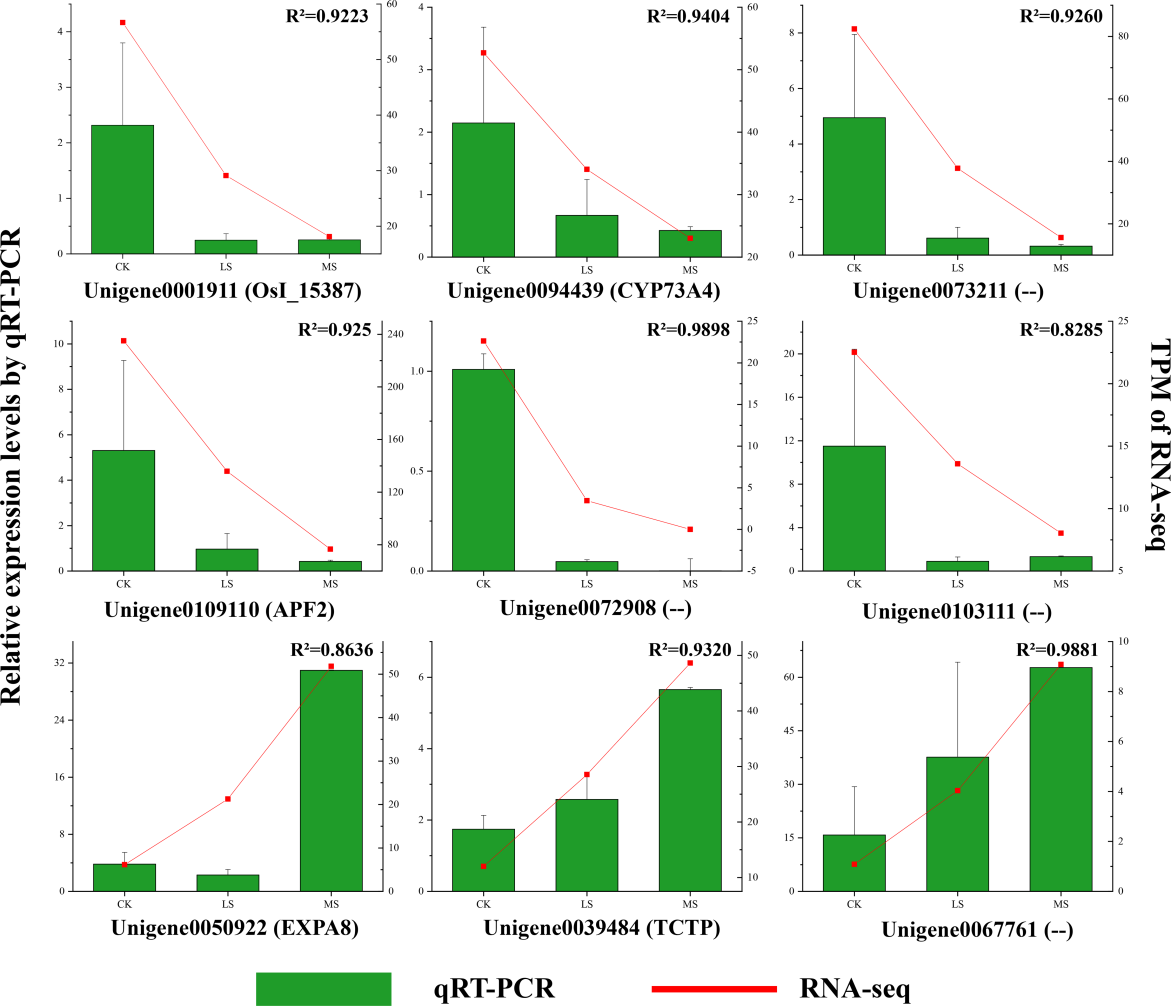
Validation of selected hub genes from RNA-seq results by qRT-PCR. Relative expression levels under CK, control (0 ‰ NaCl); LS, low salt (3 ‰ NaCl); MS, moderate salt (5 ‰ NaCl) conditions. RNA-seq values are shown as transcripts per million (TPM). Error bars represent ± SE of three biological replicates.

To validate the transcriptome sequencing results, nine genes were randomly selected from the hub genes for qRT-PCR analysis. The expression trends obtained from qRT-PCR were in good agreement with the TPM values (**Figure 6**). Further correlation analysis demonstrated a correlation coefficient of R^2^ ≥ 0.8285, indicating that the transcriptome data were highly accurate and reliable.

## 4 Discussion

It is well documented that salt stress has a detrimental effect on the growth and development of crops, with the potential to result in their death (Lv et al., 2012). As time progresses and salinity levels increase, there is an observable increase in the magnitude of biomass reduction and leaf damage become more pronounced (Saha et al., 2015). In a similar manner, for *T. mucronatum*, elevated salt concentrations had been demonstrated to inhibit growth, with substantial leaf damage being observed at 5 ‰ salinity and plant death at 7 ‰. Consequently, it can be deduced that the salt stress tolerance threshold for *T. mucronatum* is 5 ‰. Soils exhibiting an electrical conductivity (EC) of ≥2 dS m^-1^ are traditionally considered saline-alkali soils (Hassani et al., 2021), and thus *T. mucronatum* possesses the potential for application on such soils.

Membrane stability techniques have been extensively used in the evaluation of the responses of various plant genotypes to salt stress, thereby facilitating the assessment of salt tolerance among different genotypes (Mahlooji et al., 2018). As Farooq and Azam (2006) demonstrated, alterations to salinity could also effect changes to the saturation and fluidity of membrane fatty acid. In accordance with the findings of studies conducted on crops such as wheat and maize (Esfandiari and Gohari, 2017; Chen et al., 2018), the present study found that as salt concentration increased, the contents of REL and MDA dose-dependently rose. This finding indicated an increased level of leakage and intensified lipid peroxidation of cell membranes, which directly reflected the damage caused by salt ions to the cellular components of *T. mucronatum*.

To mitigate further damage from salt ions, plants must readjust their physiological and biochemical processes and reconfigure their growth and development paradigms that involve restoring osmotic and ionic homeostasis (Zhang et al., 2022). In this study, we found that the contents of PRO, SS, and SP in *T. mucronatum* accumulated significantly. These osmoregulatory substances are crucial for regulating cellular osmotic pressure and alleviating the adverse effects of salt stress (Bose et al., 2014). In addition to osmoregulation, the antioxidant enzyme system, a vital stress resistance mechanism in plants, also plays a key role in scavenging ROS generated under salt stress (Xia et al., 2014), as evidenced by the increasing activities of POD, CAT, and SOD in *T. mucronatum*. Notably, we observed that PRO content and SOD activity showed no significant differences between the LS treatment and CK treatment, while they were significantly elevated in the MS treatment. PRO is considered to play a significant role in inducing plant tolerance to stress conditions (Liang et al., 2013), and high levels of proline can also serve as a nitrogen source during plant recovery (Ali et al., 2007). SOD activity is detected in nearly all plant cell types and catalyzes the conversion of singlet oxygen to molecular oxygen and hydrogen peroxide (Hu and Schmidhalter, 2005). The differential performance exhibited at different salt concentrations is considered a specific manifestation of *T. mucronatum*, namely, a graded antioxidant strategy against high salt concentrations (Li et al., 2021). Similarly, in plants treated with polyethylene glycol (PEG), significant differences in PRO content were observed only when PEG concentration exceeded 20% (Wu et al., 2022). In sugar beet plants, SOD activity did not show significant changes at low concentrations, but only increased significantly at concentrations above 140 mM (Wang et al., 2017). Furthermore, PRO is capable of promoting the activities of antioxidant enzymes like SOD (Devnarain et al., 2016). Therefore, when the salt concentration reaches 5 ‰, the increase in PRO content and SOD activity is crucial for withstanding higher stress levels.

Through transcriptomic analysis, we found that the number of DEGs in the MS treatment was significantly lower compared to the CK treatment, especially among the upregulated genes. This observation is consistent with the findings in *Nitraria sibirica* (Li et al., 2021), where a similar pattern of more DEGs responding to lower salt concentrations was observed. However, in the study of *Limonium bicolor* (Zhu et al., 2025), higher salt concentrations consistently led to a greater number of DEGs. We propose that these differences are related to the genotype of each species, as each species exhibits distinct responses to varying salt concentrations. For *T. mucronatum* and *N. sibirica*, the strong salt tolerance of these species may result in the upregulation of a large number of genes under low-salt treatment, thereby enhancing their adaptability to salt stress (Li et al., 2021).

To further elucidate the functional implications of these DEGs, we conducted GO enrichment analysis. Our results revealed that the upregulated DEGs were significantly enriched in terms related to oxidoreductase activity, a finding that mirrors observations in *Glycine max* (Fu et al., 2024). This suggests that *T. mucronatum* activates relevant genes to enhance oxidoreductase activity. Consequently, the activities of POD, CAT, and SOD were elevated, thereby bolstering defense mechanisms against salt stress. In contrast, the downregulated DEGs were primarily associated with the “cell wall organization or biogenesis” pathway. Salt stress is known to induce cell wall damage, leading to its softening and remodeling, as well as alterations in membrane fluidity, integrity, and function. These changes can result in the production of damage-associated molecular patterns, which in turn modulate stress signaling (Saijo and Loo, 2020). The increase in MDA and REL indicates escalating stress damage in *T. mucronatum*. In response to salt stress, plants have been observed to modulate the synthesis and deposition of major cell wall components. This process has been shown to mitigate damage, prevent water loss, and reduce the transport of residual ions into the plant tissues (Dabravolski and Isayenkov, 2023).

KEGG analysis showed that the DEGs were mostly enriched in pathways like Biosynthesis of various plant secondary metabolites, Phenylpropanoid biosynthesis, and Carotenoid biosynthesis, echoing the pattern seen in Chenopodium quinoa under salt stress (Li and Zhang, 2025). This underscores the critical role of plant metabolic and synthetic processes in responding to salt stress. Notably, distinct enrichment profiles emerged in *Populus* (Liu et al., 2025b) and *Nitraria sibirica* (Li et al., 2021) when subjected to different salt concentrations. In the CK vs. LS comparison, pathways like Photosynthesis and Flavonoid biosynthesis were enriched, a finding that resonates with observations in *Limonium bicolor* (Zhu et al., 2025). This highlights the importance of photosynthetic optimization and flavonoid metabolism in plant responses to salt stress. In contrast, under higher salt stress, pathways such as Monoterpenoid biosynthesis and Linoleic acid metabolism were enriched. This further reinforces the idea that metabolic pathways and biosynthetic processes are among the most representative responses to salt stress (Lei et al., 2021).

Increasing evidence has established the crucial roles of transcription factors (TFs) in regulating diverse biological processes, including responses to abiotic and biotic stresses (Ng et al., 2018). Among these, TF families such as AP2/ERF, WRKY, and bHLH are key players in mediating responses to abiotic stresses, including salt stress (Chen et al., 2023). Notably, across both monocot and dicot plants, numerous ERF genes exhibit significant upregulation in response to salt stress (Bahieldin et al., 2016; Li et al., 2018; Wessels et al., 2019). For instance, overexpression of the *TaERF3* in transgenic wheat plants has been shown to enhance their adaptability to salt stress (Rong et al., 2014). In our study, we also observed that in both the CK vs. LS and CK vs. MS treatment comparisons, the ERF family was the most prominently represented among TF families involved in salt stress response (**Figure 3G**). This suggests that ERFs play a vital role in the salt stress response of *T. mucronatum*, a finding that aligns with previous reports (Xu et al., 2013).

Through WGCNA, we identified six hub genes. Among these, four (*PGL3*, *OsI_15387*, *APF2*, and *CYP73A4*) exhibited progressive downregulation with increasing salt stress concentration, while two (*TCTP* and *ECI3*) showed consistent upregulation. Plant cell wall biosynthesis is a complex, tightly regulated process. Salt stress can severely impact cell wall biosynthesis, thus disrupting normal plant growth and development (Dabravolski and Isayenkov, 2023). Pectin, a major component of the cell wall, is crucial for maintaining cell integrity, mediating defense responses, and facilitating cell adhesion (Bethke et al., 2016). Polygalacturonases (PGs) are the principal pectin hydrolase involved in cell wall degradation and remodeling (Ke et al., 2018). Additionally, lignin is a key structural component of plant cell walls, and its reduced synthesis can compromise cell wall strength (Coomey et al., 2020). The sustained decrease in expression levels of *PGL3* (*Unigene0103111*) and *CYP73A4* (*Unigene0094439*) suggests that salt stress may suppress cell wall metabolism and growth activities, thereby affecting cell wall stability and integrity. This aligns with our observed increase in MDA and RCL. *OsI_15387* (*Unigene0001911*) encodes PCNT115, an auxin-induced protein implicated in auxin signaling and plant growth (Gao et al., 2014). Its downregulation implies that salt stress may inhibit auxin signal transduction and related physiological processes, potentially slowing plant growth, which matches our findings. APF2 (*Unigene0109110*), an aspartic protease family member involved in protein degradation and processing (Chen et al., 2015), is speculated to indicate suppressed protein metabolism under salt stress (Raimbault et al., 2013). These findings collectively indicate that the physiological state of *T. mucronatum* is increasingly compromised as salt concentration rises.

TCTP is a highly conserved, multifunctional protein critical for plant growth, development, and stress responses (Tao et al., 2015). *TCTP* generally acts as a positive regulator to enhance plant stress resistance in common abiotic stresses (Kim et al., 2012; Sharma et al., 2017). In this study, we found that *TCTP* was upregulated in *T. mucronatum* to resist salt stress, which was consistent with the results of *Hevea brasiliensis* (Li et al., 2013). In potato, *StTCTP* was found to be highly expressed in response to stress, and overexpression of *StTCTP* enhanced CAT enzyme activity (Liu et al., 2025a), which was in line with our observed CAT index. In biological systems, the oxidation of unsaturated fatty acids is a key metabolic process (van Weeghel et al., 2014). This process requires certain auxiliary enzymes, such as enoyl-CoA isomerases, which catalyze the isomerization of enoyl-CoA, helping unsaturated fatty acids enter the β-oxidation pathway (Poirier et al., 2006). β-oxidation is the primary pathway for fatty acid degradation and occurs in plant peroxisomes (Goepfert et al., 2008). It provides carbon and energy for plant growth through the catalytic breakdown of fatty acids (Graham, 2008). Thus, we speculate that *T. mucronatum* upregulates *ECI3* (*Unigene0045322*) to increase unsaturated fatty acid oxidation, thereby providing energy to combat salt stress. Further research is needed to confirm this function. Additionally, two unannotated genes, one hypothetical protein and three uncharacterized proteins, showed significant upregulation or downregulation under salt stress and warrant further investigation.

By superimposing the six physiological traits onto the co-expression network we obtained a systems-level view in which the accumulation of proline, soluble sugars/proteins and the elevated activities of POD, CAT and SOD map onto the MEbrown module, while their widespread repression characterizes the MEblue module (**Figure 4D**). This correlation matrix provides the missing bridge between transcriptional reprogramming and phenotypic output. Collectively, unlike model annuals, *T. mucronatum* exhibited a sharp survival threshold at 5 ‰ NaCl, beyond which irreversible leaf senescence and whole-plant death occurred within 15 d. This critical threshold, together with the identification of 12 hub genes (especially, *TCTP*, *ECI3*, *PGL3*, *OsI_15387*, *APF2*, *CYP73A4*) not previously reported in salt-stressed trees, extends our understanding of salt tolerance from herbaceous models to long-lived woody perennials. Before these hub genes and the associated MEbrown/MEblue regulatory modules can be deployed in breeding or restoration programs, however, the stability of the 5 ‰ threshold and the network topology must be confirmed by repeating the salt-gradient experiment in an independent growing season. The present study focused on discovering global regulatory nodes rather than correlating single-gene expression with specific enzyme assays; isoform-level validation can be pursued under the control of the identified hubs in future work. Meanwhile, functional confirmation of the identified hub genes is hampered by the absence of a transformation protocol for *T. mucronatum*; future heterologous transformation studies are warranted to dissect the roles of these core regulators under salt stress.

## 5 Conclusion

The present study explored the physiological and molecular mechanisms underlying salt tolerance in *T. mucronatum*, identifying a critical salinity threshold of 5 ‰ NaCl. Exceeding this level resulted in irreversible damage and plant death. The plants exhibited a progressive response: minimal osmotic adjustments under low salinity, and activated osmoprotectant and antioxidant enzyme systems under moderate salinity. The transcriptomic analysis revealed 3,858 DEGs, with oxidoreductase functions and secondary metabolite biosynthesis pathways playing central roles in adaptation. Six key regulatory genes (*TCTP*, *ECI3*, *PGL3*, *OsI_15387*, *APF2*, *CYP73A4*) were identified, contributing to stress resilience through mechanisms including cell wall modification, redox balance maintenance, and energy production regulation. This investigation provides the first transcriptomic analysis of *T. mucronatum* and enhances our understanding of stress adaptation mechanisms in woody perennials. The identification of genotype-specific tolerance thresholds and key regulatory genes offers valuable targets for the development of salt-tolerant cultivars. Furthermore, its capacity to thrive in soils with conductivity exceeding 2 dS m^-1^ positions it as a promising species for the rehabilitating of saline-alkali ecosystems.

## Data Availability

The original contributions presented in the study are included in the article/**Supplementary Material**. Further inquiries can be directed to the corresponding authors.
