# Supplementary material for: Morphological, physiological, and transcriptomic analysis of Taxodium mucronatum under different salinity stresses
Source: Front Plant Sci. 2026 Feb 4;17:1686191. doi: 10.3389/fpls.2026.1686191 (PMC12913384; doi:10.3389/fpls.2026.1686191)
Supplement: Supplementary file 1 [file DataSheet1.docx]

Supplementary Material


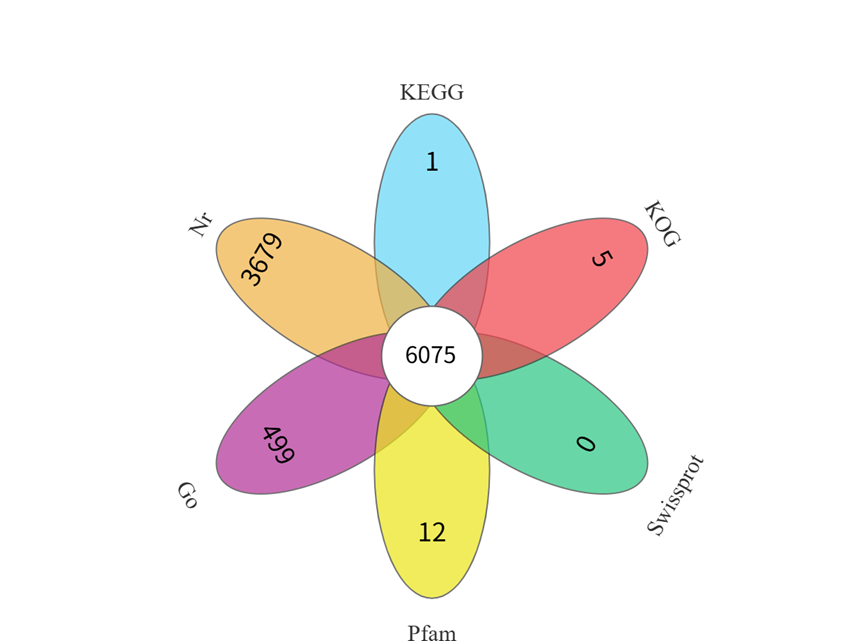


**Supplementary Figure 1.** Venn diagram of unigene annotations across six databases.

# Supplementary Table 1. Primer sequences for qRT-PCR analysis of selected genes.

| Primer | Sequence (5' to 3') | Base Number |
| --- | --- | --- |
| Uniggene0001911-F | GGACTAGGGATTCTGAGGCC | 20 |
| Uniggene0001911-R | GCTCCTGAGGCAAAGAAACC | 20 |
| Uniggene0094439-F | TGGTAGGAGAAGTTGCCCAG | 20 |
| Uniggene0094439-R | TCGAAGTTCTGAACCAGCCT | 20 |
| Uniggene0073211-F | CACATCCCTCCAAATGCACC | 20 |
| Uniggene0073211-R | TCCACCGACTTCACTTCCAA | 20 |
| Uniggene0109110-F | GTCAAACGATGCCAACCCTT | 20 |
| Uniggene0109110-R | TAATCGTCCCATCTCCGCTC | 20 |
| Uniggene0072908-F | GGATGCAAGGCGAGAAAGAG | 20 |
| Uniggene0072908-R | ACACGATGGTAGGCGAGAAA | 20 |
| Uniggene0103111-F | TACGCAGACCAGTCCAATGT | 20 |
| Uniggene0103111-R | CGTTGTCGGCATAGGAGTTG | 20 |
| Uniggene0050922-F | GAGACGTTCATGCTGTGTCC | 20 |
| Uniggene0050922-R | CCTTGGAATGTTTGCCCGAA | 20 |
| Uniggene0039484-F | TCGAAGTGAACATTGGAGCG | 20 |
| Uniggene0039484-R | ACTGGTTCATCATCTACGCCT | 21 |
| Uniggene0067761-F | GGCCACGTTACACCTTCTCT | 20 |
| Uniggene0067761-R | ACACAACCCACATCGGTCTC | 20 |
| GAPDH-F | TGTCCCCAAGGGTTTCACTG | 20 |
| GAPDH -R | GTATCCACCACTGCTTCCCA | 20 |

# Supplementary Table 2. Evaluation statistics of sequencing data.

| Sample | Raw Reads | Clean Reads | Q20 (%) | Q30 (%) | GC Content (%) |
| --- | --- | --- | --- | --- | --- |
| CK-1 | 38519426 | 38373382 | 98.78% | 96.28% | 43.94% |
| CK-2 | 41169778 | 41011356 | 98.87% | 96.51% | 44.03% |
| CK-3 | 40821072 | 40663268 | 98.96% | 96.80% | 43.94% |
| LS-1 | 37177924 | 36972508 | 98.49% | 95.52% | 43.47% |
| LS -2 | 41791026 | 41605506 | 98.69% | 96.05% | 43.32% |
| LS -3 | 41913664 | 41755254 | 98.87% | 96.53% | 43.39% |
| MS-1 | 41501046 | 41336262 | 98.87% | 96.54% | 43.33% |
| MS -2 | 44496300 | 44342570 | 98.83% | 96.34% | 43.35% |
| MS -3 | 36887250 | 36751464 | 98.87% | 96.49% | 43.57% |
